# Pharmacist intervention through protocol-based pharmacotherapy management is effective to ensure safety in invasive procedures for chronic liver disease

**DOI:** 10.1186/s40780-025-00456-z

**Published:** 2025-08-14

**Authors:** Yuuka Shibata, Yuki Koga, Yuki Sato, Emiko Mashida, Tomokazu Kawaoka, Eisuke Murakami, Kei Amioka, Yusuke Johira, Kensuke Naruto, Takanori Taogoshi, Tomoharu Yokooji, Masataka Tsuge, Hiroaki Matsuo

**Affiliations:** 1 Department of Pharmaceutical Services, Hiroshima University Hospital, 1–2–3 Kasumi, Minami-Ku, Hiroshima, Japan; 2 Department of Gastroenterology, Graduate School of Biomedical and Health Sciences, Hiroshima University, 1–2–3 Kasumi, Minami-Ku, Hiroshima, Japan; 3 Department of Frontier Science for Pharmacotherapy, Graduate School of Biomedical and Health Sciences, Hiroshima University, 1–2–3 Kasumi, Minami-Ku, Hiroshima, Japan

**Keywords:** Chronic liver disease, Protocol-based pharmacotherapy management, Platelet products, Thrombocytopenia, Thrombopoietin receptor agonist

## Abstract

**Background:**

The risk of hemorrhagic complications in patients with chronic liver disease during invasive procedures should be considered. Patients with low platelet counts were administered platelet products prior to procedures based on a physician's judgment. However, there are no standards for allocating the bleeding risk associated with each procedure, platelet counts to avoid these risks, or methods for determining platelet counts. In this study, we evaluated whether pharmacists could reduce the use of platelet products by suggesting thrombopoietin receptor agonists using protocol-based pharmacotherapy management to assess procedural bleeding risk and platelet counts.

**Methods:**

Among patients with chronic liver disease who were scheduled to undergo invasive procedures between August 2022 and February 2023, those who were interviewed by a pharmacist prior to the procedures were defined as the intervention group (*n* = 80) and the others as the non-intervention group (*n* = 224). The protocol was to define the procedural bleeding risk and platelet count. Pharmacists suggested prescribing a thrombopoietin receptor agonist to patients with platelet counts below the recommended counts.

**Results:**

The use of platelet products and thrombopoietin receptor agonists was 0% and 7.5% and 3.1% and 0% in the intervention and non-intervention groups, respectively. Among the patients who were required to receive lusutrombopag, all patients in the intervention groups did not receive platelet product but lusutrombopag alone. However, the rates of patients with the recommended platelet count were not different between the intervention and non-intervention groups.

**Conclusions:**

The use of platelet products decreases without the increased incidences of hemorrhage if pharmacists suggest prescribing thrombopoietin receptor agonists based on their assessment of the platelet count and the bleeding risk of the procedure.

## Background

Patients with chronic liver disease (CLD) have a reduced platelet generation capacity due to bone marrow suppression and decreased thrombopoietin (TPO) production in the liver. Platelet counts gradually decrease due to progressive liver damage and platelet destruction [1]. Patients with CLD typically undergo invasive procedures several times a year for diagnosis and treatment, and these procedures involve bleeding risk. Severe bleeding during invasive procedures without treatment for thrombocytopenia can easily lead to hemorrhagic complications and prolonged hospitalization. The risks associated with spontaneous bleeding, such as coagulopathy and esophageal/gastric varices, should also be considered before the procedure [2, 3]. In these respects, management of bleeding risk related to thrombocytopenia is an important issue for patients with CLD [4].

There are several treatment options for thrombocytopenia, including splenectomy, platelet transfusion, and the administration of TPO receptor agonists. Although platelet-based products are frequently used to treat thrombocytopenia, they have several disadvantages. First, there are adverse effects such as fever, urticaria, anaphylaxis, transfusion-related acute lung injury, and other serious complications [5, 6]. Additionally, repeated administration may cause platelet transfusion refractoriness due to anti-human leukocyte antigen (HLA) antibodies [7]. These adverse reactions may result in prolonged hospitalization, increased bleeding risk, and higher medical costs. Second, platelet products are blood donor-derived drugs that are sometimes in short supply. Because of these disadvantages, the guidelines for platelet transfusion usage indicate that platelet products should be used minimally, as needed [8]. Therefore, several guidelines recommend using TPO receptor agonists as an alternative to platelet products [4, 9, 10].

Treatment is clinically decided by a physician based on the platelet count and bleeding risk of the procedure being performed. However, there are no practical guidelines for choosing treatment based on the bleeding risk of the procedures and platelet counts. Alvaro et al. [11] reported that no clear platelet count was required for any invasive procedure. Therefore, the use of platelet products and TPO receptor agonists has not yet been standardized.

We developed a protocol for the recommendation of lusutrombopag, a TPO receptor agonist, based on the bleeding risk for each invasive procedure and the platelet count for protocol-based pharmacotherapy management (PBPM) [12]. Pharmacists interviewed patients with CLD scheduled for invasive procedures to assess bleeding risk and platelet counts and suggested prescribing lusutrombopag according to the protocol. In this study, we evaluated whether PBPM could reduce the use of platelet products in patients with CLD.

## Methods

### Protocol development

Based on a report by Yoshiji et al*.* [13], we categorized the risk levels for each procedure and defined the platelet count required for each level. The degree of bleeding risk was defined based on mild risk, including transcatheter arterial chemoembolization (TACE), endoscopic variceal ligation (EVL), abdominocentesis, balloon-occluded retrograde transvenous obliteration (BRTO), and computed tomography (CT) during arterial portography (CTAP). Moderate risks included radiofrequency ablation (RFA), endoscopic injection sclerotherapy (EIS), endoscopic submucosal dissection (ESD), endoscopic mucosal resection (EMR), liver biopsy (BX), liver tumor biopsy (TmBX), microwave ablation (MWA) and percutaneous ethanol injection therapy (PEIT).

**Fig. 1:**
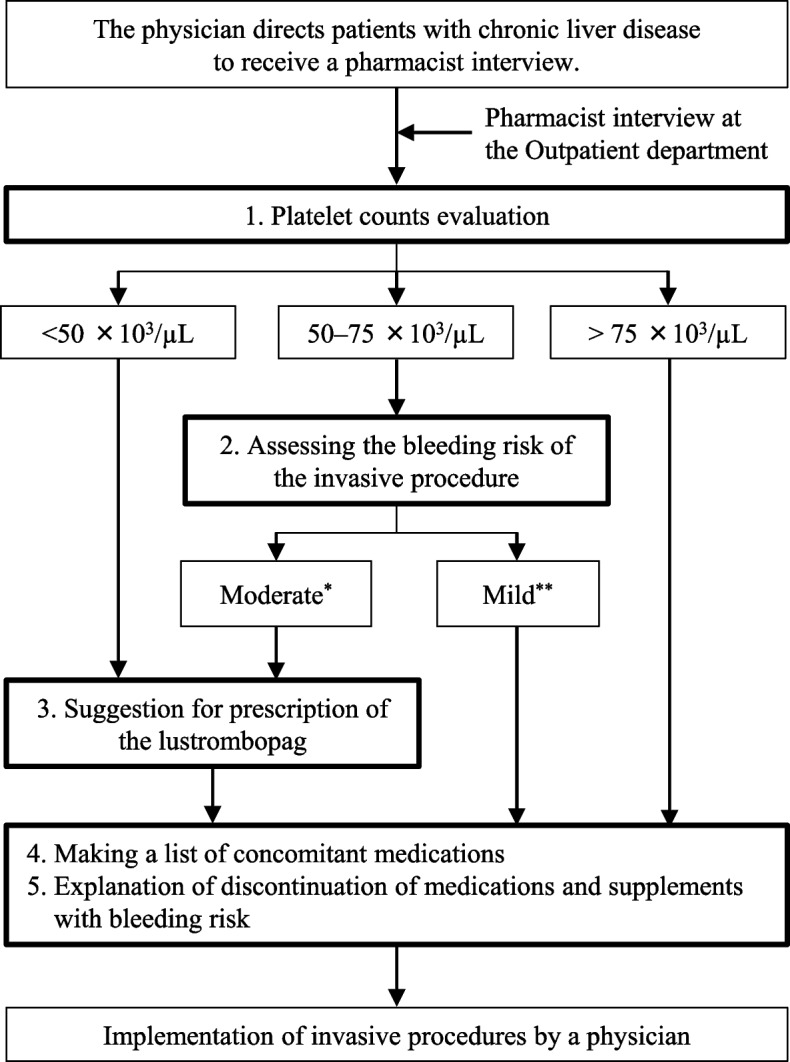
Flowchart of a pharmacist interview to suggest a prescription for a TPO receptor agonist. *The moderate risk includes Radiofrequency ablation (RFA), Esophageal varices sclerotherapy (EIS), Submucosal dissection (ESD), Mucosal resection (EMR), Liver biopsy (BX), Liver tumor biopsy (TmBX), Microwave thermal coagulation (MWA) and Percutaneous ethanol injection therapy (PEIT). **The mild risk includes Chemical embolization of the hepatic artery (TACE), Esophageal vein ligation (EVL), Ascites puncture, Retrograde transverse vein thrombolysis under baroreflex occlusion (BRTO), Computed tomography under portal venography (CTAP)

We developed a protocol to determine whether lusutrombopag administration should be recommended (Fig. 1). Briefly, pharmacists classified the patients by the platelet count into three categories: < 50 × 10^3^/µL, 50–75 × 10^3^/µL, and > 75 × 10^3^/µL. Pharmacists suggested prescribing the lusutrombopag for all patients with platelet counts < 50 × 10^3^/µL and for patients with platelet counts of 50–75 × 10^3^/µL undergoing invasive procedures with the moderate bleeding risk as described above. These two suggestions were based on the package insert for lusutrombopag (Mulpleta® Tablets), the former based on clinical trial data, and the latter defined by the statement “This agent should be used when the patient is considered to be at high risk of bleeding based on platelet counts and other laboratory data, clinical symptoms, and the type of invasive procedure performed”. Yoshiji et al*.* reported that a platelet counts of at least 75 × 10^3^/µL is desirable for patients undergoing moderate-risk procedures [13]. Therefore, we recommended lusutrombopag even if the platelet count was above 50 × 10^3^/µL. This protocol-based pharmacist intervention was performed when the pharmacist interviewed patients with CLD in the outpatient department. The interviews included checking platelet counts, assessing procedural bleeding risk, suggesting the use of lusutrombopag based on the protocol, listing medications prescribed by other hospitals and clinics and supplements and/or over-the-counter medications in use, extracting medications and supplements with bleeding risks, such as antithrombotic agents, and explaining their discontinuation. The medication lists of patients who were not directed to be interviewed by a pharmacist in the outpatient department were prepared by a ward pharmacist through an interview after admission.

### Patients

Patients with CLD who were scheduled to undergo invasive procedures at the Department of Gastroenterology and Metabolism at Hiroshima University Hospital between August 2022 and February 2023 were enrolled. The exclusion criteria were as follows: patients who had participated in a clinical trial, patients who had undergone or are undergoing a liver transplant, patients with portal vein tumor thrombosis, adult-onset-type citrullinemia, aplastic anemia, myelodysplastic syndrome, myelofibrosis congenital thrombocytopenia, drug-induced thrombocytopenia, immune thrombocytopenia, generalized infection requiring treatment except for viral liver disease, and Child–Pugh class C. The physicians requested an interview with the pharmacist if the patient was taking medications at the time of their examination and the patient has sufficient time for the interview. Patients who were directed to be interviewed by a pharmacist were defined as the pharmacist's intervention (Ph-intervention) group, whereas others were defined as the non-intervention group.

### Data collection and analysis

The data used in this study were obtained from clinical records. We extracted data on patient age, sex, type of chronic liver disease, Child–Pugh classification, degree of hepatic encephalopathy and ascites, bilirubin level, albumin level, prothrombin activity level, type of invasive procedure, baseline platelet count, use of medications, use of over-the-counter drugs, use of supplements with bleeding risk, occurrence of thrombosis and bleeding during hospitalization, lusutrombopag prescription, and administration of platelet products. As the primary endpoint in this study, effectiveness was compared between the rate of platelet administration prior to the procedure in the Ph-intervention and non-intervention groups. Safety was evaluated by comparing the numbers of adverse and thrombotic events. In a subgroup analysis, the rate of platelet product administration, platelet counts at the time of the invasive procedure and the incidence of thrombotic events in the patients who were required to receive lusutrombopag were compared between the two groups.

### Statistical analysis

Characteristics were compared between the Ph-intervention and non-intervention groups and analyzed using the Mann–Whitney U test for continuous variables and Fisher's exact test for categorical variables. Differences in the rate of platelet product use and the incidence of thrombotic events were compared between the two groups using Fisher's exact test. Univariate logistic regression analysis was used to calculate the odds ratios (ORs) and 95% confidence interval (95% CI) of thrombosis cases in the Ph-intervention and non-intervention groups. Differences were considered statistically significant at *P* < 0.05. Data analyses were performed using the JMP pro version 17.0.0 (SAS Institute, Cary, NC, USA).

### Ethics statement

This study was approved by the Ethics Committee of Hiroshima University (approval No. E2022-0220) in compliance with the Declaration of Helsinki and current legal regulations in Japan. Before collecting the data, we provided the participants with the opportunity to opt out. Information collected from medical records was entered into a case report form and de-identified by an information manager. Therefore, the researchers were unable to identify individuals from the analyzed data.

## Results

### Patient characteristics

**Fig. 2:**
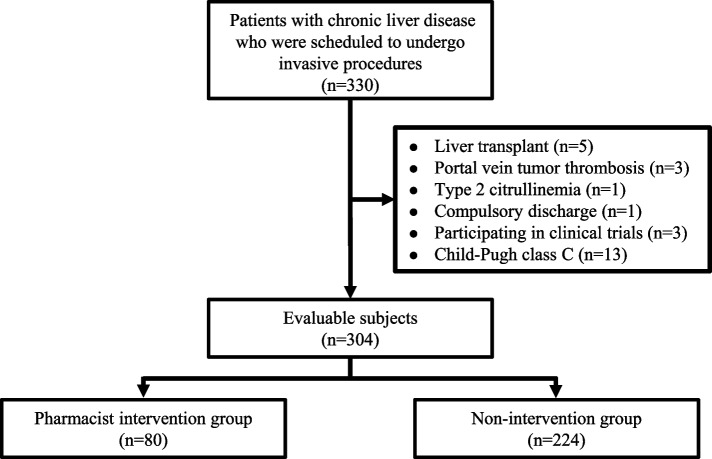
Flowchart of patient recruitment

The procedure for participant enrollment is shown in Fig. 2. After excluding 26 cases according to the criteria, 304 cases were included in the analysis, of which 80 received pharmacist intervention in the outpatient department (Ph-intervention group) and the remaining 224 did not (non-intervention group).

The characteristics of patients with CLD are shown in Table 1. The median age of patients was 75 ± 12.4 years in the Ph-intervention group and 75 ± 9.7 years in the non-intervention group. There was no significant difference in the age distribution between the two groups. There were also no significant differences in the type of CLD or Child–Pugh classification between the groups. However, the proportion of males was higher in the non-intervention group (74.0%, 166/224) than in the Ph-intervention group (60.0%, 48/80). Additionally, the number of moderate risks in the invasive procedure was significantly higher in the Ph-intervention group than in the non-intervention group. There were no significant differences in the proportions of the baseline platelet counts for mild and moderate risk and in the median baseline prothrombin activity between the two groups. The bleeding-risk medications listed at the time of the interview included aspirin, rivaroxaban, ethyl icosapentate, prasugrel, omega-3-acid ethyl esters, clopidogrel, dilazep, dabigatran, cilostazol, edoxaban, limaprost, and warfarin. Supplements with bleeding risk were docosahexaenoic acid and/or eicosapentaenoic acid, saw palmetto, ibuprofen and ginseng. These medications and supplements were discontinued before the invasive procedure, with the exception of aspirin in two patients. The minor bleeding events were mild puncture site bleeding and nose bleeding. No patients had prolonged hospitalization due to adverse events such as hemorrhage and thrombotic events.

**Table 1:** Characteristics of patients with chronic liver disease

		Ph-intervention (*n* = 80)		Non-intervention (*n* = 224)		*p*-value
Median age, years (IQR)		75 (63–80)		75 (69–81)		0.065
Gender, *n* (%)						
Male		48	(60)	166	(74)	0.018
Female		32	(40)	58	(26)	-
Type of CLD, *n* (%)						
Hepatitis B		7	(9)	25	(11)	0.546
Hepatitis C		25	(31)	78	(35)	0.562
Other		48	(60)	121	(54)	0.355
Child–Pugh class, *n* (%)						
A		63	(79)	155	(69)	0.103
B		17	(21)	69	(31)	-
Invasive procedure, *n* (%)						
Moderate risk*		45	(56.3)	47	(21.0)	< 0.001
Mild risk**		35	(43.8)	177	(79.0)	-
Baseline platelet count (× 10^3^/μL)						
Moderate risk						
< 50		1	(2.2)	1	(2.1)	0.975
50–74		3	(6.7)	8	(17.0)	0.126
≧75		41	(91.1)	38	(80.9)	0.158
Mild risk						
< 50		2	(5.7)	4	(2.3)	0.260
50–74		7	(20.0)	19	(10.7)	0.127
≧75		26	(74.3)	154	(87.0)	0.055
Median baseline prothrombin activity, percentage (IQR)						
		88 (32–116)		84 (34–121)		0.293
Use of platelet-related products, *n* (%)						
Platelet products		0	-	7	(3.1)	-
Lusutrombopag		6	(7.5)	0	-	-
Taking anti-thrombotic agents, *n* (%)						
Yes		15	(18.8)	65	(29.0)	0.073
No		65	(81.3)	159	(71.0)	-
Taking supplements or OTC with bleeding risk, *n* (%)						
Yes		2	(2.5)	5	(2.2)	0.89
No		78	(97.5)	219	(97.8)	-
Events, *n* (%)						
Hemorrhage***		0	-	5	(2.2)	-
Thrombosis		1	(1.3)	1	(0.5)	0.445

Data are presented as medians with IQR or n (%)

*CLD* chronic liver disease, *IQR* interquartile range, *OTC* over the counter medicine, *Ph* pharmacist

^*^Moderate-risk includes radiofrequency ablation (RFA), esophageal varices sclerotherapy (EIS), submucosal dissection (ESD), mucosal resection (EMR), liver biopsy (BX), liver tumor biopsy (TmBX), microwave thermal coagulation (MWA) and percutaneous ethanol injection therapy (PEIT)

^**^Mild risk includes Chemical embolization of the hepatic artery (TACE), Esophageal vein ligation (EVL), Ascites puncture, Retrograde transverse vein thrombolysis under baroreflex occlusion (BRTO), Computed tomography under portal venography (CTAP)

^***^The hemorrhage events were puncture site bleeding (*n* = 3) and nose bleeding (*n* = 2). Statistical significance was set at *P* < 0.05

### Patients who were recommended to receive the lusutrombopag

Of the seven patients who were recommended lusutrombopag based on the protocol in the pharmacist interview, six were prescribed it. There were two BX cases, two EIS cases, one EVL case, and one TACE case. The platelet counts of five patients who were at a moderate risk of bleeding increased to > 75 × 10^3^/µL while that of one patient at a mild bleeding risk increased to > 50 × 10^3^/µL. All six patients underwent invasive procedures without hemorrhage and the other events, and no adverse effects were reported after taking lusutrombopag. The seventh patient was not prescribed the lusutrombopag because the procedure they were to undergo was mild risk EVL. The patient's platelet count was maintained at 49 × 10^3^/µL and at the time of the procedure was 66 × 10^3^/µL. The procedure was performed without bleeding. In contrast, there were patients who would have qualified for lusutrombopag prescription in the non-intervention group if they had followed the protocol, but none of them were prescribed, including five who received platelet products.

### Patients receiving platelet products

The use of platelet products is shown in Table 2. Usage was 0% (0/80) in the Ph-intervention group and 3.1% (7/224) in the non-intervention group. The univariate logistic regression analysis showed lower usage of platelet products in the Ph-intervention group compared to the non-intervention group (*p* = 0.19; Table 2). No anaphylaxis or other adverse reactions to the platelet products were observed. Of the seven patients who received platelet products, five had a moderate bleeding risk. Even though the platelet counts were between 50–75 × 10^3^/µL and were eligible for lusutrombopag treatment based on the protocol, they were not prescribed. Two of the seven patients underwent a procedure with a mild bleeding risk. Their platelet counts were above 50 × 10^3^/µL and they were therefore not eligible for taking the lusutrombopag, but neither had stopped taking aspirin because of prevention of cardiovascular disease recurrence.

**Table 2:** Usage of platelet products

	Platelet product use		*p*-Value
	No	Yes	
Non-intervention (*n* = 224)	217	7 (3.1%)	
Ph-intervention (*n* = 80)	80	0 (0%)	0.19

Data are presented as *n* (%). Ph, pharmacist

### Incidence of thrombotic events

The incidence of thrombotic events is shown in Table 3. The incidence rates were 1.23% (1/80) and 0.45% (1/224) in the Ph-intervention and non-intervention groups, respectively. Univariate logistic regression analysis showed no significant difference in the incidence between the two groups by increasing lusutrombopag usage (OR: 2.82 [95% CI: 0.17–45.7], *p* = 0.47; Table 3).

**Table 3:** Incidence of the thrombotic events

	Thrombosis episode		*p*-Value	OR (95% CI)
	No	Yes		
Non-intervention (*n* = 224)	223	1 (0.45%)		1 (Ref.)
Ph-intervention (*n* = 80)	79	1 (1.23%)	0.46	2.82 (0.17–45.7)

Data are presented as *n* (%)

*Ph* pharmacist, *OR* Odds ratio, *CI* Confidence interval

### Analysis in patients who were required to receive lusutrombopag

The rate of platelet product use, platelet counts at the time of the invasive procedure and the incidence of thrombotic events in patients who were required to receive lusutrombopag were analyzed. In the intervention group, no patients (0/6) received a platelet product without lusutrombopag whereas all patients (7/7) in the non-intervention groups received a platelet product. The rate of patients who had the recommended platelet count at the time of the invasive procedure was not different between the intervention group (83%, 5/7) and the non-intervention group (57%,4/7, *p* = 0.56; Table 4). No thrombotic events occurred in either group.

**Table 4:** Subgroup analysis in patients who were required to receive lusutrombopag

Platelet product use			
	No	Yes	*p*-Value
Non-intervention (*n* = 7)	0	7 (100%)	
Ph-intervention (*n* = 6)	6	0 (0%)	0.001
Patients with the recommended platelet counts at the time of the invasive procedure			
	No	Yes	*p*-Value
Non-intervention (*n* = 7)	3	4 (57%)	
Ph-intervention (*n* = 6)	1	5 (83%)	0.56

Data are presented as *n* (%). Statistical significance was set at *P* < 0.05. Ph, pharmacist

## Discussion

Despite the importance of preventing hemorrhagic complications associated with thrombocytopenia during invasive procedures in patients with CLD, practical guidance is insufficient in clinical practice. In this study, we developed and implemented a protocol to avoid bleeding risks and ensure the safe performance of the procedures. Our new protocol focuses on four key points: 1) defining each invasive procedure as mild or moderate risk according to the degree of bleeding risk; 2) classifying the patient's platelet counts into three categories; 3) recommending TPO receptor agonists instead of platelet products; and 4) determining the combination of the procedure's bleeding risk and the patient's platelet counts for screening patients who should take TPO receptor agonists. Among the patients who were required to receive lusutrombopag, the rate of patients with the recommended platelet count at the time of the invasive procedure did not differ between the intervention and the non-intervention groups although the rate of the use of a platelet product was lower in the intervention group than in the non-intervention groups. Furthermore, no thrombotic events occurred in either group. These results suggest that using this protocol through interviews with patients with CLD prior to admission, pharmacists could contribute to increasing TPO receptor agonist prescriptions and decreasing platelet product usage.

In this study, there were more cases with mild bleeding risk in the non-intervention group, in contrast to more cases with the moderate risk in the Ph-intervention group (Table 1). This could be because moderate-risk bleeding procedures for patients taking antithrombotic medications increase mortality risk; thus, physicians might have wanted pharmacists to intervene in risk management. The primary purpose of the pharmacist interview was to list the medications being taken and check for bleeding risk medications, including supplements. In other words, physicians may not request a pharmacist interview with a patient who has no medications at the time of the physician's examination. Additionally, if the patient did not have sufficient time for the interview, the interview was not requested. Therefore, mild-risk procedures may have been more frequent in the non-intervention group. This could lead to a bias in the background between the two groups. However, subgroup analysis showed no difference in platelet counts between the two groups even though patients in the intervention group underwent more higher-risk procedures than those in the non-intervention group and did not receive platelet product.

There were more cases of hemorrhage in the non-intervention group, but not in the intervention group (Table 1). The five hemorrhage cases were minor bleeding that did not require transfusion, such as mild puncture site bleeding and nose bleeding. None of these patients had prolonged hospitalization and their platelet counts, and prothrombin activity were within reference range. Furthermore, there was no difference in baseline platelet counts and prothrombin activity between these two groups. Therefore, these events were considered incidental events in the non-intervention group with a large number of patients.

In the Ph-intervention group, which included many patients at a moderate risk of bleeding, no patients received platelet products (Table 2). In addition, none of the six patients who were prescribed lusutrombopag instead of platelet products experienced any adverse events (Table 3). In contrast, in the non-intervention group, 13 patients were eligible for lusutrombopag prescriptions, including five patients who received platelet products. These five patients could have avoided receiving platelet products through pharmacist intervention. Platelet products are recommended for minimal use because of complications, distribution problems, and religious reasons [5, 6, 8]. In particular, urgent recommendations regarding the proper use of blood products were issued in 2020, owing to the shortage of blood products caused by Covid-19, and more proper use was requested in Japan. In Europe, the use of TPO receptor agonists is recommended as an alternative to platelet products [4, 9]. Our protocol prioritizes TPO receptor agonists in the treatment of thrombocytopenia and avoids the use of platelet products in patients with CLD. The advantages of TPO receptor agonists are as follows: (1) platelet counts are easy to predict, (2) patients can take them home, (3) they are easy to store, and (4) they are not susceptible to supply shortages. Platelet products are recommended for patients with Child–Pugh Classification C, children, and pregnant women who cannot receive TPO receptor agonists. The use of these products may also be considered in patients whose platelet counts do not sufficiently increase with TPO receptor agonists. These patients were excluded from the study. Further studies are required to evaluate the efficacy of this protocol in a larger number of patients.

The criteria for lusutrombopag administration in this protocol require further discussion. In the non-intervention group, 13 patients had low platelet counts, but were not prescribed lusutrombopag. Eight did not receive platelet products but the procedure was performed without bleeding events. The consensus guideline of the Central European Hepatologic Collaboration (CEHC) guideline development group recommends that liver biopsy should be performed with a platelet count of at least 50 × 10^3^/µL, whereas our protocol set this recommendation to greater than 75 × 10^3^/µL [9]. This is because liver biopsy is a diagnostic examination, and priority was placed on minimizing the occurrence of hemorrhagic complications. In addition, two patients received platelet products despite their platelet counts being above 50 × 10^3^/µL. In this case, the patient was receiving aspirin, and the products were administered to account for the risk of bleeding due to its antiplatelet effects. This suggests that the protocol should reflect not only the platelet count but also the bleeding risk caused by medications such as antithrombotic agents. Further studies are needed to determine the optimal combination of platelet count and procedural bleeding risk in patients for whom TPO-receptor agonists should be prescribed.

This study had several limitations. First, we did not investigate the effects of other TPO receptor agonists. Two oral TPO-receptor agonists, avatrombopag and lusutrombopag, are available in Japan. However, avatrombopag was not commercially available at the beginning of this study. Secondly, the sample size was small, especially for patients with a platelet count of less than 75 × 10^3^/µL who underwent moderate-risk procedures. The rate of these patients was not significantly different between the non-intervention and intervention groups (19.1% vs. 8.9%), but the small number of cases may be a factor in the non-significance of this difference. Therefore, it could not be denied that this difference in patient background led to a difference in the use of platelet products because many patients in the non-intervention group used platelet products. Larger studies are needed to analyze the impact of different patient backgrounds and the presence of patients who are resistant to treatment with TPO receptor agonists. Thirdly, there may have been bias in patient background due to the physician's judgment in assigning patients to each group. As a general rule, risk management to avoid bleeding risks is the highest priority. However, if the physician determined that the pharmacist interview cannot be conducted due to lack of patient time or no medication to be taken, the patients will be divided into the non-intervention group.

## Conclusions

The pharmacist assessed the bleeding risk of the procedure based on the protocol evaluated in this study and recommended the use of lusutrombopag according to the platelet count, resulting in a reduced use of platelet products. This may have contributed to improved safety for the patients with CLD in this study who underwent invasive procedures.

## Data Availability

All data generated or analyzed during this study are included in this published article.

## Abbreviations

BRTO: Balloon-occluded retrograde transvenous obliteration
BX: Liver biopsy
CI: Confidence interval
CLD: Chronic liver disease
CTAP: Computed tomography during arterial portography
EIS: Endoscopic injection sclerotherapy
EMR: Endoscopic mucosal resection
ESD: Endoscopic submucosal dissection
EVL: Endoscopic variceal ligation
HLA: Anti-human leukocyte antigen
IQR: Interquartile range
MWA: Microwave ablation
OR: Odds ratio
PBPM: Protocol-based pharmacotherapy management
PEIT: Percutaneous ethanol injection therapy
Ph: Pharmacist
RFA: Radiofrequency ablation
TACE: Transcatheter arterial chemo embolization
TmBX: Liver tumor biopsy
TPO: Thrombopoietin
